# Revealing the Relevant Spatiotemporal Scale Underlying Whole-Brain Dynamics

**DOI:** 10.3389/fnins.2021.715861

**Published:** 2021-10-22

**Authors:** Xenia Kobeleva, Ane López-González, Morten L. Kringelbach, Gustavo Deco

**Affiliations:** ^1^Department of Neurology, University of Bonn, Bonn, Germany; ^2^Computational Neuroscience Group, Department of Information and Communication Technologies, Center for Brain and Cognition, Universitat Pompeu Fabra, Barcelona, Spain; ^3^German Center for Neurodegenerative Diseases (DZNE) Bonn, Bonn, Germany; ^4^Department of Psychiatry, University of Oxford, Oxford, United Kingdom; ^5^Centre for Eudaimonia and Human Flourishing, University of Oxford, Oxford, United Kingdom; ^6^Department of Clinical Medicine, Center for Music in the Brain, Aarhus University, Aarhus, Denmark; ^7^Institució Catalana de la Recerca i Estudis Avançats (ICREA), Barcelona, Spain; ^8^Department of Neuropsychology, Max Planck Institute for Human Cognitive and Brain Sciences, Leipzig, Germany; ^9^School of Psychological Sciences, Monash University, Clayton, VIC, Australia

**Keywords:** modeling, spatiotemporal, brain dynamics, functional connectivity, brain networks

## Abstract

The brain rapidly processes and adapts to new information by dynamically transitioning between whole-brain functional networks. In this whole-brain modeling study we investigate the relevance of spatiotemporal scale in whole-brain functional networks. This is achieved through estimating brain parcellations at different spatial scales (100–900 regions) and time series at different temporal scales (from milliseconds to seconds) generated by a whole-brain model fitted to fMRI data. We quantify the richness of the dynamic repertoire at each spatiotemporal scale by computing the entropy of transitions between whole-brain functional networks. The results show that the optimal relevant spatial scale is around 300 regions and a temporal scale of around 150 ms. Overall, this study provides much needed evidence for the relevant spatiotemporal scales and recommendations for analyses of brain dynamics.

## Introduction

The brain is a dynamical system that can process and adapt to new information by rapidly transitioning between multiple states. Human functional neuroimaging studies have demonstrated these transitions of functional states, even in absence of an active task (Tang et al., 2012; Stitt et al., 2017; Liégeois et al., 2019). These functional states contain orchestrated activity of several networks of brain regions, which transition between each other in recurring patterns over time (Alexandrov, 1999; Ashourvan et al., 2017; van der Meer et al., 2020). These network transitions have been associated with cognition and (ab)normal behavior (Engel et al., 2001; Bassett et al., 2011; Thompson et al., 2013; Vidaurre et al., 2017; Liégeois et al., 2019; Yoo et al., 2020). While describing these transitions is important for our understanding of the neural basis of cognition and behavior, a fundamental question remains, namely on which spatiotemporal scale (i.e., spatiotemporal resolution) these transitions between whole-brain functional networks generally take place. This unclarity has led to arbitrary decisions in neuroimaging experiments regarding spatiotemporal scales, risking losing relevant information on network transitions.

The main reason for a lack of empirical evidence for the optimal spatiotemporal scales of brain transitions is caused by technical restrictions of human neuroimaging studies. Despite a coherent overlap between functional networks in EEG/MEG and fMRI, i.e., EEG microstates and fMRI resting state networks (Britz et al., 2010; Musso et al., 2010; Yuan et al., 2012), these neuroimaging modalities differ regarding the available spatial and temporal scale. The spatial scale of MEG depends on the number of sensors, and beamforming can only separate up to around 70 regions across the whole brain with a significant drop in signal in deeper regions (Brookes et al., 2016; Deco et al., 2017a). Regarding the temporal scale, EEG and MEG provide a temporal scale of milliseconds, whereas fMRI is limited by the repetition time (TR) and the hemodynamics of the BOLD signal. In line with the respective strengths of the modalities, spatial scales and parcellations have been mainly explored with fMRI (Proix et al., 2016; Arslan et al., 2018), while temporal scales of brain dynamics have been rather investigated using EEG or MEG (Bassett et al., 2006; Britz et al., 2010). However, current technical restrictions make simultaneous comparisons of spatial and temporal scales insurmountable.

One way of overcoming the technical limitations of spatiotemporal scales of empirical neuroimaging data is to use generative dynamical network models that simulate whole-brain neural activity in a simplified manner, while retaining the main properties of empirical spatiotemporal dynamics. In a broad sense, generative models conceptualize the relevant underlying processes (e.g., synaptic connectivity and dynamics of neural populations) that generate the observed empirical neural signal, e.g., by using mean-field approximations (Wilson and Cowan, 1972; Stephan et al., 2007; Deco et al., 2013). In contrast to restricted spatiotemporal scales of empirical data, these models recover the underlying neural signal with a flexible adaptation of the signal’s temporal scale up to a millisecond scale (Yuan et al., 2018; Deco et al., 2019; Cornblath et al., 2020), while simultaneously offering an exploration of various spatial scales (Gerstner et al., 2012).

In our previous research, we were able to compare the complexity of dynamic transitions of whole-brain networks using a whole-brain network model incorporating different temporal scales from milliseconds to seconds (Deco et al., 2019). In the current study, we extend this approach by adding a spatial scale to the analysis (i.e., the number of regions) and exploring the transitions of networks across different spatiotemporal scales (i.e., simultaneously considering spatial and temporal scales). The novelty of our study comes from the information about the optimal number of brain regions (i.e., spatial scale) in describing the spatiotemporal dynamics of the resting-state, providing an empirical basis for the choice of parcellations for analyses of brain dynamics. By doing so, we complement previous neuroimaging studies on the temporal scale of functional whole-brain networks’ transitions (e.g., Bassett et al., 2006) and the spatial scale of static networks (e.g., using functional connectivity as in Arslan et al., 2018).

To achieve our goal, we explore transitions of whole-brain functional networks at spatial scales from 100 to 900 regions. To add a temporal dimension, these networks are extracted from empirical resting-state fMRI with fixed temporal scales (corresponding to the TR) as well as from simulated time series with various temporal scales from milliseconds to seconds. We determine the relevant spatiotemporal scale by comparing the entropy of functional network transitions. By focusing on the behavior of whole-brain networks instead of separate brain regions, we extract information that is the most relevant for brain dynamics analyses. From our results we derive recommendations for neuroimaging researchers, highlighting our finding that the optimal spatial scale for analyses of brain dynamics is at around 300 regions and at an optimal temporal scale of around 150 ms. Besides implications for a better understanding of functional network transitions, we provide data-based recommendations for choosing appropriate neuroimaging modalities and parcellation techniques for brain dynamics analyses.

## Materials and Methods

We adapted the existing method comparing different time scales (Deco et al., 2019) to incorporate different spatial scales. Images were created using Biorender, Inkscape, Connectome Workbench and the Matplotlib library within Python.

### Data Acquisition and Preprocessing

We used the 100 unrelated subjects’ subset (54 females, 46 males) from the Human Connectome Project (HCP) (Van Essen et al., 2013). From this data, we analyzed the left-right (LR) phase-encoding runs from the resting state fMRI data, which had about 15 min duration with a TR of 0.72 sec. The HCP study was approved by the local ethical committees and informed consent was obtained from all subjects. Data from six subjects were discarded as the resulting functional connectivity (FC) matrices consisted of at least one not available row at parcellations with more than 800 regions (due to the sparsity of the networks), resulting in a total of 94 subjects being used for the analysis. During fMRI acquisition, subjects were instructed to keep their eyes open while looking at a fixation cross. A full description of the imaging parameters and minimal preprocessing pipeline can be found in Glasser et al. (2013). In short, after correction for motion, gradient, and susceptibility distortions the fMRI data was aligned to an anatomical image. The aligned functional image was then corrected for intensity bias, demeaned, and projected to a common surface space, which resulted in a cifti-file.

All fMRI data were filtered between 0.1 and 0.01 Hz to retain the relevant frequency range for further analyses of the BOLD signal. We obtain structural and functional matrices in different spatial scales using the Schaefer parcellation, which optimizes local gradient and global similarity measures of the fMRI signal in various spatial scales ranging from 100 to 900 regions (Schaefer et al., 2018). These parcellations can be found on https://github.com/ThomasYeoLab/CBIG/tree/master/stable_projects/brain_parcellation/Schaefer2018_LocalGlobal. In both fMRI datasets time series were extracted with the help *Workbench Command* provided by the HCP.

To create a structural connectome as a basis for the whole-brain model, we generated an average structural connectome depicting the number of fibers in the required spatial scales. We used an independent diffusion MRI dataset from the HCP database with a subset of 32 participants acquired at the Massachusetts General Hospital (“MGH HCP Adult Diffusion,” 16 females, 16 males). This dataset had been acquired using high-quality scanning protocols (e.g., using an optimized scanner to achieve a high *b*-value of 10,000 s/mm^2^ and a high-angular resolution, combined with a multi-slice approach), resulting in above-average normative diffusion MRI data. The data had already been preprocessed and made available to the public within the Lead-DBS software package (Setsompop et al., 2013; Horn et al., 2017). In brief, the data were processed using a generalized q-sampling imaging algorithm as implemented in *DSI studio*.^1^ The data were segmented and co-registered using *SPM 12*. Restricted by a coregistered white-matter mask, 200,000 fibers were sampled within each participant using a Gibbs’ tracking approach (Kreher et al., 2008) and normalized into MNI space via DARTEL transforms (Ashburner, 2007; Horn and Blankenburg, 2016). We used the standardized methods from *Lead-DBS toolbox* version 2.0 (Horn et al., 2018) to obtain structural connectomes for the same parcellation schemes as for the functional data, selecting tracts that both started and ended within the specified parcellation scheme.

### Whole-Brain Modeling Using the Dynamic Mean Field Model

The use of fMRI signals would normally limit our study in the temporal dimension. To overcome this shortcoming, we use a whole-brain dynamic mean field (DMF) model, which allows us to simulate data in varying timescales from milliseconds to seconds, while maintaining a comparable structure of the signal. The model expresses the activity of interconnected brain regions with excitatory and inhibitory influences as a reduced set of coupled stochastic differential equations (Deco et al., 2013), following the original derivation by Wong and Wang (2006). Thus, this model allows us to describe the activity of brain regions on a macroscale, while considering the dynamics of local neuronal pools (i.e., inhibitory and excitatory neural populations). Importantly, considering these local dynamics, the model gives a description of local dynamics at millisecond scales. This fine-grained temporal scale cannot be achieved with other whole-brain dynamical models, which usually simulate oscillatory activity in the same temporal scale as the empirical signal (e.g., bifurcation-based models as in Deco et al., 2017b).

**FIGURE 1:**
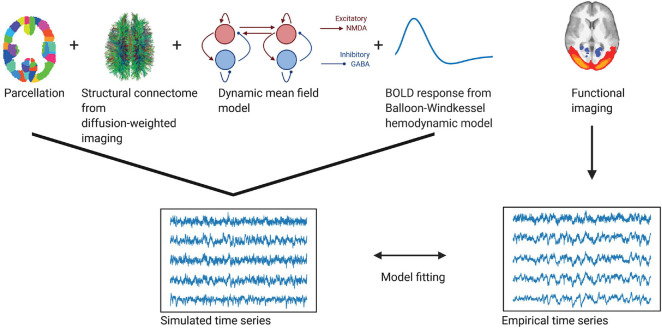
Whole-brain modeling steps to create simulated functional time series fitted to empirical BOLD data. Using a whole-brain network model such as the dynamic mean field model allows us to accurately create time series data at different temporal scales. Local dynamics of each region given by a *parcellation* are generated by a *dynamic mean field model* and coupled through the *structural connectome* (as provided by the numbers of fiber tracts estimated from diffusion-weighted imaging). To fit the resulting neuronal time series to the empirical BOLD time series, we employ a *Balloon-Windkessel hemodynamic model* to create simulated BOLD time series. The simulated time series are *fitted* to the empirical time series using metrics of metastability and phase similarity matrix distributions.

A summary of the individual steps to create the model can be found in Figure 1. The model consists of a network of brain regions that emit spontaneous neuronal signals. The spatial scale defines the number of the brain regions. Each of these regions consists of excitatory (*E*) and inhibitory (*I*) neuronal pools, which reciprocally influence each other locally within each region *n*. We further assume that these regions interact via long-range connections, as given by the connection weights of the structural connectome (Deco et al., 2014).

We implement equations based on these assumptions in a modified DMF model, which is based on the original reduction first proposed by Wong and Wang (2006). In the model used in this study, NMDA receptors mediate excitatory currents *I*^(*E*)^ and GABA-A receptors mediate inhibitory currents *I*^(*I*)^. Inhibitory sub-populations communicate reciprocally with excitatory sub-populations on a local level. Excitatory sub-populations are additionally linked to other excitatory sub-populations via long-range connections, representing the effect of NMDA receptors. These long-range connections *C* are based on the number of fiber tracts given by the structural connectome (see description above). The connections are then tuned by the global scaling factor *G* that linearly scales all synaptic strengths.

The following set of coupled differential equations are used to create the DMF model:


(1) $$\begin{array}{c} I_{n}^{\left(E\right)}=w_{E}I_{0}+w+w_{NMDA}S_{n}^{\left(E\right)}+Gw_{NMDA}\Sigma_{p}C_{np}S_{p}^{\left(E\right)}-w_{n}S_{n}^{\left(I\right)} \end{array}$$

(2) $$\begin{array}{c} I_{n}^{\left(I\right)}=w_{I}I_{0}+w_{NMDA}S_{n}^{\left(E\right)}-S_{n}^{\left(I\right)} \end{array}$$

(3) $$\begin{array}{c} r_{n}^{\left(E\right)}=H^{\left(E\right)}\left(I_{n}^{\left(E\right)}\right)=\frac{g_{E}\left(I_{n}^{\left(E\right)}-I_{thr}^{\left(E\right)}\right)}{1-\text{exp}\left(-d_{E}g_{E}\left(I_{n}^{\left(E\right)}-I_{thr}^{\left(E\right)}\right)\right)} \end{array}$$

(4) $$\begin{array}{c} r_{n}^{\left(I\right)}=H^{\left(I\right)}\left(I_{n}^{\left(I\right)}\right)=\frac{g_{I}\left(I_{n}^{\left(I\right)}-I_{thr}^{\left(I\right)}\right)}{1-\text{exp}\left(-d_{I}g_{I}\left(I_{n}^{\left(I\right)}-I_{thr}^{\left(I\right)}\right)\right)} \end{array}$$

(5) $$\begin{array}{c} \frac{dS_{n}^{\left(E\right)}\left(t\right)}{dt}=-\frac{S_{n}^{\left(E\right)}}{\tau_{NMDA}}+\left(1-S_{n}^{\left(E\right)}\right)\gamma r_{n}^{\left(E\right)}+\sigma \upsilon_{n}\left(t\right) \end{array}$$

(6) $$\begin{array}{c} \frac{dS_{n}^{\left(I\right)}\left(t\right)}{dt}=-\frac{S_{n}^{\left(E\right)}}{\tau_{GABA}}+r_{n}^{\left(I\right)}+\sigma \upsilon_{n}\left(t\right) \end{array}$$

For each inhibitory (*I*) and excitatory (*E*) neuronal pool in every brain region *n*, the vector $I_{n}^{\left(E,I\right)}$ represents the total input current (in nA), the vector $r_{n}^{\left(E,I\right)}$ stands for the firing rate (in Hz) and the vector $S_{n}^{\left(E,I\right)}$ denotes the synaptic gating. The total input currents, which are received by the neuronal pools, are converted by the neuronal response functions *H*^(*E*,*I*)^ into firing rates $r_{n}^{\left(E,I\right)}$. Here, the gain factors *g_E_*=310 nC^−1^ and *g_I_*=310 nC^−1^ are used to determine the slope of *H*. The shape of the curvature of H around *I*_*thr*_ is defined by the constants *d_E_* = 0.16 and *d_i_*=0.087. When the threshold currents of $I_{thr}^{\left(E\right)}$=0.403 nA and $I_{thr}^{\left(I\right)}$=0.288 nA are reached, the firing rates increase linearly with the input currents. We add a standard Gaussian noise υ_*n*_ with an amplitude of σ = 0.01 nA (Deco et al., 2014).

The average synaptic gating of the excitatory pools $S_{n}^{\left(E\right)}$ is controlled by the NMDA receptors with a decay time constant τ_*NMDA*_ = 0.1 s and γ = 0.641 (transformed into ms). The average synaptic gating of the inhibitory pools $S_{n}^{\left(I\right)}$ is controlled by the GABA receptors with a decay time constant τ_*GABA*_ = 0.01 s (transformed into ms).

Synaptic currents *I_n_* result from inputs from the local network, i.e., $S_{n}^{\left(E,I\right)}$, and inputs from other network nodes, i.e., $S_{p}^{\left(E\right)}$. Local inputs are governed by weights, i.e., *w_E_*, *w*_*NMDA*_, *w*_*n*_, *w*_*I*_ and *w*. All excitatory synaptic couplings are weighted by *w*_*NMDA*_ = 0.15 and the weight of the recurrent excitation *w* 1.4. Additionally, there is a constant input to each neural pool, denoted by *I_0_* = 0.382 nA with the weights *w_E_* = 1 and *w_I_* = 0.7. The local parameters are chosen so that the average firing rate is close to 3 Hz (Deco et al., 2014). Only the weight of feedback inhibition *w_n_* is adjusted before each simulation using a regulatory mechanism called Feedback Inhibition Control, which mimics a resting state condition (Deco et al., 2014). This ensures that the network stays in its asynchronous state with firing rates between 3 and 10 Hz for all regions.

Inputs from other regions $S_{p}^{\left(E\right)}$ are given by the excitatory populations and are weighted by the connection weights of the structural connectome *C*_*np*_. These connection weights are scaled by the global coupling parameter *G*, which is adjusted using model fitting (see below).

It is then possible to retrieve separate temporal scales from the simulated neuronal data by binning the time series. However, first the neuronal time series had to be fitted to the empirical BOLD time series (by adjusting *G*) to ensure a biologically plausible signal. Therefore, we transformed the neuronal signal from the model into a simulated BOLD signal and then compared the simulated and empirical signals (see below). We employed the Balloon-Windkessel hemodynamic model using all biophysical parameters as stated in Stephan et al. (2007). The model is described by the following equations:


(7) $$\begin{array}{c} \frac{ds_{n}}{dt}=0.5r_{n}^{\left(E\right)}+3-ks_{n}-\gamma \left(f_{n}-1\right) \end{array}$$

(8) $$\begin{array}{c} \frac{df_{n}}{dt}=s_{n} \end{array}$$

(9) $$\begin{array}{c} \tau \frac{dv_{n}}{dt}-f_{n}-v_{n}^{a^{-1}} \end{array}$$

(10) $$\begin{array}{c} \tau \frac{dq_{n}}{dt}=\frac{f_{n}{\left(1-\rho \right)}^{f_{n}^{-1}}}{\rho }-\frac{q_{n}v_{n}^{a^{-1}}}{v_{n}} \end{array}$$

This model describes a vasodilatory signal *s_n_* which is altered by autoregulatory feedback. Depending on *s_n_*, the blood flow *f_n_* leads to changes of the deoxyhemoglobin content *q_n_* and blood volume *v_n_*. τ is the time constant, ρ is the resting oxygen fraction and *a* represents the venous resistance. For each region *n* the BOLD signal *B*_*n*_ is a static non-linear function of *q_n_* and *v_n_*:


(11) $$\begin{array}{c} B_{n}=V_{0}\left[k_{1}\left(1-q_{n}\right)+k_{2}\left(1-\frac{q_{n}}{v_{n}}\right)+k_{3}\left(1-v_{n}\right)\right] \end{array}$$

To focus on the functionally relevant frequency range, we band-pass filtered the simulated BOLD signals using the same filter as for the empirical data with a bandpass between 0.1 and 0.01 Hz (Achard et al., 2006; Glerean et al., 2012). A summary of the model parameters can be found in Supplementary Table 1.

### Agreement Between Empirical and Simulated Data

To achieve biologically plausible signal statistics in the simulated time series at each scale, we performed the fitting to the empirical signals by adjusting G to have a maximal agreement in three different metrics: the metastability, phase consistency matrices and functional connectivity dynamics (see below). Each of these metrics represent different dynamical properties of the BOLD signal. Previous research has shown that adding these dynamical metrics such as metastability and phase consistency matrices to the fitting procedures improves constraining dynamical working points of dynamical whole-brain models than using only static metrics such as FC (Deco et al., 2017b, 2019; Saenger et al., 2017). These metrics were computed for each value of G (between 0 and 2.5 in steps of 0.025) in the simulated data and for the empirical data and compared as described below. Due to multiple spatial scales, the creation of the model was very compute-intensive, e.g., to replicate the time series of 10 subjects from the HCP dataset at a neuronal timescale using a parcellation of 400 regions with different *G*-values from 0 to 2.5 about 80–100 GB of RAM and 30 days of computation were required (mostly owing to the feedback inhibition control mechanism). Therefore, we restricted the simulations to 10 iterations, representing the time series of a group of 10 subjects. To ensure that our analyses were generalizable to different healthy subjects, we did 100 iterations of the model fitting to empirical time series with a random selection of 10 subjects at each iteration.

#### Dynamical Measures Used for the Fitting

##### Metastability

The metastability represents the overall variability of oscillations (Wildie and Shanahan, 2012; Deco et al., 2017b). It is calculated as the standard deviation of the Kuramoto order parameter R(t) across time, which depicts the average phase φ*_*k*_*(*t*) in each region *k* across *n* regions.


(12) $$\begin{array}{c} R\left(t\right)=\frac{\left|\sum_{k=1}^{n}e^{i\varphi_{k}\left(t\right)}\right|}{n} \end{array}$$

The phases were derived from the data by detrending the filtered fMRI time series and then applying the Hilbert transform. When *R* = 1, all phases are fully synchronized, while *R* = 0 indicates a complete desynchronization of all phases. We calculated the differences between the empirical and simulated metastability. This has been previously proven to be suitable to define the dynamical working point of dynamical whole-brain models (Deco et al., 2017b; Saenger et al., 2017).

##### Phase Consistency Matrices

We calculated the phase coherence matrix by evaluating the instantaneous phase at each time point *t* of every region *j* and then computing the phase difference across all regions. We measured the similarity of these phase coherence matrices over *t* to create a *phase consistency matrix*. This matrix is a representation of spatiotemporal fluctuations of phases. To compare empirical and simulated data, we calculated the Kolmogorov-Smirnov distance between the empirical and simulated distribution of the phase consistency matrices. The Kolmogorov-Smirnov distance quantifies the maximal difference between two distribution functions of two samples and is minimized by the optimal value of G (Saenger et al., 2017).

##### Functional Connectivity Dynamics

We split the BOLD signal into sliding windows of 80 s, overlapping by 40 s. For each window, at time t, we calculated the FC, FC(t). The Functional connectivity dynamics (FCD) is defined by the Pearson correlation of the upper triangular parts of the two FC matrices FC(t1) and FC(t2). For comparison between empirical and simulated data, we calculated the Kolmogorov-Smirnov distance (see above).

Furthermore, we checked whether we retrieved comparable numbers of functional networks in the empirical and simulated data (see Supplementary Figure 2).

### Extraction of Whole-Brain Functional Networks Using Independent Component Analysis and Calculation of Entropy

**FIGURE 2:**
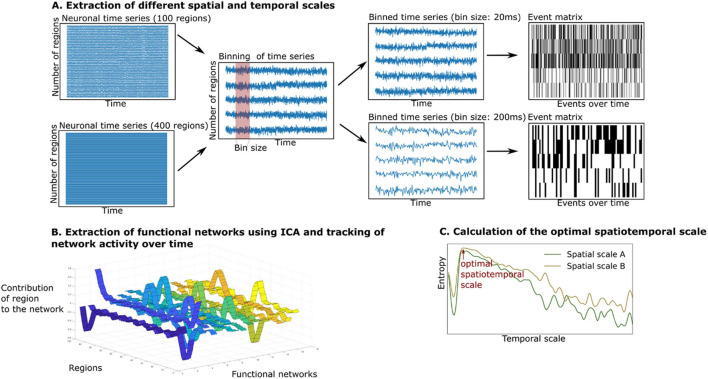
Extraction and tracking of whole-brain functional networks at different spatial and temporal scales using the whole-brain model. **(A)** We simulate neuronal time series at different spatial scales (from 100 to 400 regions). We then create different bin sizes of the time series (using bins from 10 to 3,000 ms), the bin size corresponds to the temporal scale. The binned time series are binarized using a point process paradigm, resulting in an event matrix. **(B)** We extract whole-brain functional networks using independent component analysis, resulting in a network matrix (see ribbon plot). These networks are tracked over time by projecting the event matrix onto the networks, resulting in an activity matrix (not displayed). **(C)** The richness of the switching between functional networks is estimated by calculating the entropy of their switching probability. The entropy is compared across spatial and temporal scales.

The summary of the analytical steps can be seen in Figure 2. The simulated and empirical time series were available in different spatial scales. In the case of the simulated signal, we retrieved the simulated neuronal time series at separate temporal scales in the range of milliseconds to seconds (see Figure 2A, middle panel). To do so, the simulated neuronal time series were binned by averaging the signals in windows of the width of the timescale, each time bin corresponding to a time point of the newly created time series. As this approach led to multiple fine-grained time series with a high computational cost of the analysis, we were only able to simulate the time series across all temporal scales up to a spatial scale of 400 regions. We created simulated time series at the group level by performing 10 iterations (representing 10 subjects).

In the case of the empirical time series, we extracted a group of 10 subjects from the data by randomly selecting 10 subjects. We concatenated their time series to retrieve functional networks on a group level (using the same group size as in the simulation to ensure comparability). To make the analysis robust to interindividual variability, we repeated this process 100 times. The temporal scale of the empirical data was determined by the TR (HCP: 720 ms). As only one temporal scale was provided in the empirical data, we were able to extract functional networks in a spatial scale from 100 to 900 regions.

In each temporal scale (given by the TR in the empirical data or the bin size in the simulated data), the time series were binarized using the point-process binarization algorithm for BOLD signals (Tagliazucchi et al., 2012). Here, the time series were normalized using a *z*-score transformation. Depending on the threshold, which was defined by one standard deviation, the time series were set to 0 or 1, resulting in an event matrix (see the right panel of Figure 2A), following the point-process procedure described in previous studies (Tagliazucchi et al., 2012; Deco and Kringelbach, 2017; Deco et al., 2019). Next, the event matrix was normalized using z-score transformation, so that the event matrix in each brain region would have null mean and unitary variance. This procedure has been shown to be robust for threshold choices and is a classical method to reduce dimensionality of dynamical data (Tagliazucchi et al., 2012). We then continued the analysis with the normalized event matrix ***E*** (with the dimensions: number of regions *i* x number of time points *b*).

To estimate the number of functional networks, we applied an adaptation of an eigenvalue analysis to assess the statistical significance of resulting networks (Peyrache et al., 2010; Deco et al., 2019), as introduced by Lopes-dos-Santos et al. (2013). This method finds the number of principal components within the event matrix with significantly larger eigenvalues than a normal random matrix that follows a probability function, as specified in Marèenko and Pastur (1967). As can be seen in Figure 2B, after determining the number of functional networks, we extracted these functional networks by applying an independent component analysis to the event matrix ***E***. This procedure resulted in the network matrix ***W*** (with dimensions: number of brain regions *i* x functional networks *c*).

Lastly, we tracked the activity of the functional networks over time (see Figure 2B). By projecting the binarized event matrix onto the network matrix, the similarity between each functional network *c* and the whole-brain activity at each time point *b* could be assessed. This projection resulted in an activity matrix ***A*** (with the dimensions: functional networks *c* x time points *b*):


(13) $$\begin{array}{c} A_{cb}=\vec{E_{b}^{T}}P_{c}\vec{E_{b}} \end{array}$$

with the event matrix ***E*** and the projection matrix ***P***. The projection matrix ***P*** is defined as:


(14) $$p_{c}={\vec{w}}_{c}⊗{\vec{w}}_{c}={\vec{w}}_{c}{\vec{w}}_{c}^{T}$$

where ⊗ is the outer product operator, ${\vec{w}}_{c}$ is one of the extracted functional networks from the event matrix (the column of the matrix ${\vec{w}}_{c}$) and ${\vec{E}}_{b}$ is the *b* column of the event matrix **E** (events at time point *b*).

After retrieving the activity of each functional network over time, we calculated its probability of occurrence. We calculated the ratio of activity of each functional network in relation to overall activity (activity of all networks over time), resulting in the probability of each network *c* over time:


(15) $$\begin{array}{c} p\left(c\right)=\sum_{b}A_{cb}/\sum_{c,b}A_{cb} \end{array}$$

where *b* corresponds to each time point.

Using these probabilities, we computed the entropy of occurrence of each network *c*. The entropy represents the richness of transition activity between functional networks and it captures the diversity of states depending on their probabilities, adapted from the concept ofentropy by Shannon (1948):


(16) $$\begin{array}{c} H=-\sum_{c}p\left(c\right)\log\left(p\left(c\right)\right) \end{array}$$

As the number of functional networks increased with higher spatial scales, we normalized the entropy. The normalization was done by dividing the entropy by the logarithm of the resulting number of networks for each spatial scale. By doing so, it was possible to compare across spatial scales. We then compared the entropy of network transitions across spatial and temporal scales (see Figure 2C). We made a pairwise comparison of entropy of spatial scales using Wilcoxon tests in the empirical data and the simulated data (at the optimal temporal scale and at the temporal scale = TR).

## Results

In our study we described the optimal spatiotemporal scale that captured the highest information content about the temporal evolution of functional networks (as evidenced by the transition activity). We extracted time series at different parcellations at different spatial scales (from 100 to 900 regions) in the empirical data. Furthermore, we created a dynamic mean-field model to create time series at various temporal scales from milliseconds to seconds (Figure 1) and a spatial scale between 100 and 400 regions. We extracted functional networks from both simulated and empirical time series using independent component analysis. We then explored the probability of occurrence of these functional networks over time. We calculated the entropy of these probabilities for each network, representing the diversity of transition activity between functional networks (Figure 2). By focusing our analysis on functional networks (as opposed to region-wise time series), we ensured that the information we gained on the temporal dynamics (as measured by transition activity) was relevant for whole-brain information processing.

### Agreement Between Empirical and Simulated Data

The DMF model is a neuronal model that recreates inhibitory and excitatory synaptic dynamics (including AMPA, GABA, and NMDA receptors) following the structure given by the underlying anatomical connectivity. Using the steps detailed in Figure 1 and following the constraints of anatomical connectivity as provided by the structural connectome, we created realistic neuronal time series at the scale of milliseconds to seconds using the DMF model. To ensure the robustness of the model, we fitted the resulting simulated BOLD time series to the empirical BOLD time series based on the global coupling G, which describes the constraint of the dynamics to the anatomy (Deco et al., 2013). Here, we defined a good fitting where the differences in metastability and the Kolmogorov-Smirnov statistics of the phase consistency matrices and FCD reached a minimum (see Supplementary Figure 1). As shown in Supplementary Figure 1, the fitting resulted in an optimum at a global coupling value G between 1.55 and 1.85 (depending on the spatial scale used).

**FIGURE 3:**
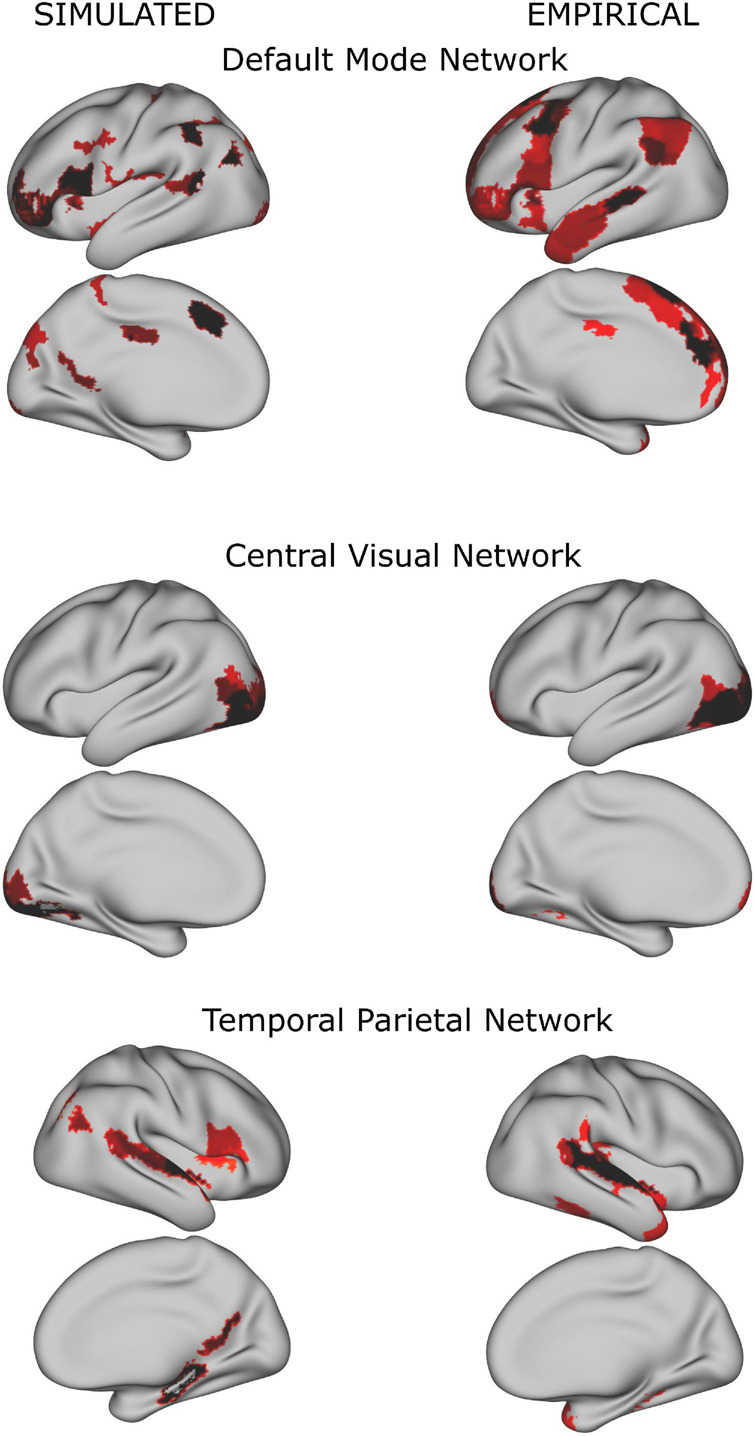
Examples of group whole-brain functional networks rendered on the standard brain. The left column has been retrieved from the simulated time series (using a TR = 720 ms), the right column from the empirical time series. Some of these networks have a high overlap with classical resting state networks (Yeo et al., 2011) such as the Default Mode Network, Central Visual Network and Temporal Parietal Network.

In both simulated and empirical data, some of the resulting networks resembled known classical resting state networks (see Figure 3). As our study focused on the dynamical alteration of functional networks, we ensured that the properties of the resulting functional networks from the simulation were comparable to the properties of the networks derived from the empirical time series. Therefore, we compared the number of functional networks derived from the simulated BOLD time series (see Supplementary Figure 2). Here, the number of functional networks increased when more regions (i.e., a higher spatial scale) were included. This finding is in agreement with the empirical data and with what was reported in the literature using similar approaches and numbers of regions (Yourganov et al., 2011; Amico and Goñi, 2018; Kumar et al., 2019).

### Entropy of Whole-Brain Functional Network Transitions

**FIGURE 4:**
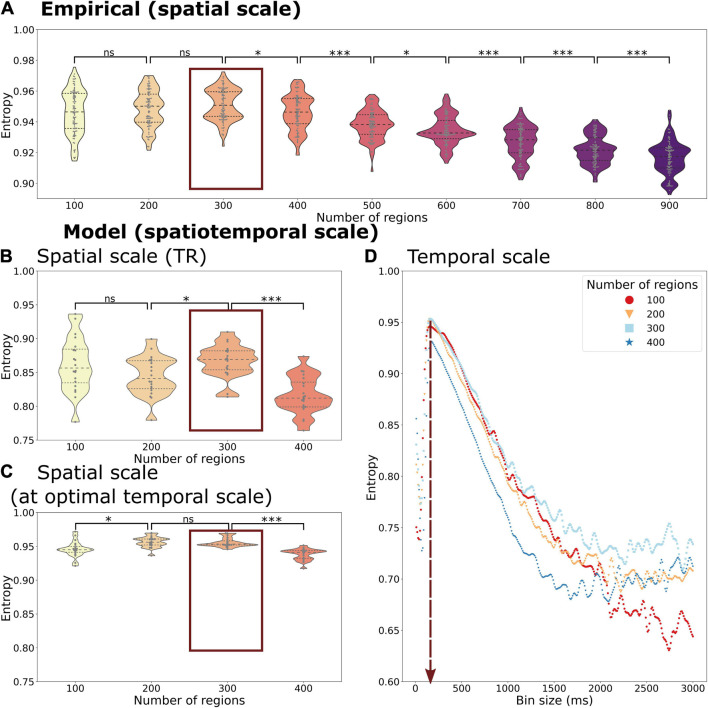
Entropy of the temporal probability of whole-brain functional networks in different spatial and temporal scales of the empirical **(A)** and simulated data **(B–D)**. The entropy is calculated across spatial scales in the empirical data with a fixed temporal scale of 720 ms (corresponding to the TR). The simulated data gives the opportunity to explore different spatial scales at the temporal scale of the TR, 720 ms, **(B)** as well as at the optimal temporal scale of 150 ms **(C)**. Beyond that it can be also used to explore various temporal scales and spatial scales simultaneously **(C)**. Both the empirical and simulated data show that the highest entropy can be found at a spatial scale of 300 regions with only a minor decrease in entropy at a spatial scale of 200 regions (marked by a red box in **A–C**). The highest entropy can be found at a temporal scale of 150 ms across all spatial scales (vertical red arrow in **D**). Each datapoint depicts a random group of 10 subjects in the empirical data or a simulation trial simulating a group of 10 subjects. Statistical significance of comparisons between spatial scales is indicated with “ns” meaning a *p* > 0.05, * meaning < 0.05, *** meaning 0.001 (FDR-corrected).

The transitions of whole-brain functional networks over time and their probability of occurrence allowed us to estimate entropy *H* as a representation of the information content of the functional network activity at various spatiotemporal scales from a probabilistic perspective. We display the entropy of spatiotemporal networks as a function of the spatial and temporal scale using empirical (Figure 4A) and simulated time series (Figure 4B).

We found an inverted U-shape form of the entropy *H* as a function of probability of spatiotemporal networks across time. Regarding the spatial scale, the *H* reached the highest value at a scale of 300 regions (mean simulated *H* = 0.957, mean empirical *H* = 0.951), but with only a small decrease at scales with 100 (mean simulated *H* = 0.949, mean empirical *H* = 0.946) or 400 regions (mean simulated *H* = 0.938, mean empirical *H* = 0.946). At spatial scales above 400 regions (analysis only present in empirical data, see Figure 4A), we observed a further drop in entropy (down to mean empirical *H* = 0.916 at 900 regions).

Beside the comparison across spatial scales, the simulated time series allowed us to compare the temporal scales (Figure 4D). Regarding the temporal scale, we found the highest entropy at an average scale of 150 ms (ranging from 140 to 160 ms, depending on the spatial scale used). Using finer or coarser temporal scales led a much greater drop in entropy (lowest value: mean simulated *H* = 0.5957) than a change of spatial scales.

Considering both spatial and temporal scales, the highest level of entropy could be found at a temporal scale of 150 ms and a spatial scale of 300 regions (see Figure 4D). The optimal temporal scale of 150 ms persisted at all simulated spatial scales. Also, the effect of temporal scale on entropy was greater than the effect of spatial scale.

Of note, *H* was always higher when using the empirical dataset in comparison to the simulated time series even when using the optimal temporal scale in the model (see Figure 4A vs. Figures 4B,C), reflecting the variability given by the empirical time series (and signals not accounted for in the DMF model).

## Discussion

In this study we investigated the most relevant spatiotemporal scale of fundamental macroscopic dynamical processes, i.e., scale of transitions between whole-brain functional networks. We followed the temporal behavior of functional whole-brain networks at different spatial scales and at fine-grained temporal scales from milliseconds to seconds (using a realistic whole-brain DMF model). Using a data-based and model-based approach, we generated evidence that the entropy of network transitions follows an inverted U-shaped curve with a maximum at a spatial scale of about 300 regions and a temporal scale of about 150 ms. Of note, the optimal temporal scale of about 150 ms persisted at all simulated spatial scales from 100 to 400 regions, indicating an absent interaction effect between spatial and temporal scales. Furthermore, the effect of the temporal scale on entropy was much greater than the effect of spatial scale, which underlines the importance of an appropriate temporal scale for analyses of brain dynamics. Given the close agreement between simulated and empirical data, the DMF model offers an excellent opportunity to bridge analyses of brain dynamics across different neuroimaging modalities and different spatiotemporal scales.

Previous studies have performed comparisons between spatial scales with various metrics, such as the reproducibility of resulting networks, agreement with anatomical connectivity, and prediction accuracy of neuropsychiatric conditions (Craddock et al., 2011; Arslan et al., 2018; Dadi et al., 2019; Messé, 2019). However, all these studies focused on the average functional connectivity, without considering network dynamics. Only Proix et al. (2016) investigated the effect of spatial scale on the information content of brain dynamics by decomposing the time series using a principal component analysis in a whole-brain network model and found the highest eigenvalue at around 140 regions. Higher spatial scales led to an oversampling with a relative reduction of connectome density, leading to more segregated regions and an overall reduction of transmission information content across regions. Although these results are promising, they focused on separate brain regions only. In contrast, we were explicitly interested in whole-brain networks, as functional networks seem to be closer related to cognitive processes than single brain regions (Dadi et al., 2019; Fan et al., 2021).

Our study is the first to examine spatial and temporal scales by simultaneously focusing on brain dynamics of whole-brain networks. Given the significant evidence that maximal entropy of brain dynamics is associated with maximal transmission of information (Lungarella and Sporns, 2006; Rämö et al., 2007; Shew et al., 2011; Wang et al., 2018), cognitive performance (Niu et al., 2018; Liu et al., 2020), and consciousness (Mashour and Hudetz, 2018), we chose to describe the richness of whole-brain network activity using the entropy of whole-brain network transitions. Selecting the most informative spatiotemporal scale during analyses of brain dynamics can help to focus the analysis on relevant information about the dynamical behavior of brain networks, while reducing the amount of noise (Fornito et al., 2010), avoiding oversampling (Proix et al., 2016), and optimizing the computational cost of the analysis, i.e., removing subnetworks that are barely active and contribute little to the overall network activity.

Our findings have several implications for future research of brain dynamics. Regarding the spatial scale, our study provides an empirical basis for choosing the number of brain regions for neuroimaging analyses that focus on brain dynamics of whole-brain functional networks. We provided evidence that a spatial scale of about 300 regions is sufficient to capture the most relevant information on brain dynamics of functional networks. While lower scales may be associated with a loss of information, higher spatial scales might introduce irrelevant and possibly more noisy functional networks.

Regarding the temporal scale, we were able to reproduce the findings of an optimal temporal scale of about 150 ms from our previous study (Deco et al., 2019), which was similar to previous simulations focusing on temporal scales of brain dynamics (Honey et al., 2007). Furthermore, our simulation results are in line with other neuroimaging findings, which showed a mean duration of EEG and MEG microstates between 100 and 200 ms (Britz et al., 2010; Baker et al., 2014). Besides a close alignment with neuroimaging studies, our findings reflect experimental results of temporal dynamics of conscious processes that operate at similar temporal scales and typically involve a rapid temporal sequence of information stabilization and transfer (Koenig et al., 2002; Van De Ville et al., 2010; Wutz et al., 2014; Salti et al., 2015; Mai et al., 2019). On top of that, our study shows that the optimal temporal scale does not depend on the spatial scale, i.e., an optimal scale of about 150 ms persists across all spatial scales. For researchers focusing on temporal properties of brain dynamics, we therefore advise to either use neuroimaging modalities operating at this optimal temporal scale (e.g., MEG or EEG, Michel and Koenig, 2018) or augment their fMRI analyses with whole-brain modeling, which allows including more fine-grained temporal scales. Therefore, based on empirical data rather than arbitrary choices, our recommendations contribute to efforts directed at harmonizing analyses of brain dynamics across spatiotemporal scales. In addition, our results underline the utility of dynamical whole-brain models to overcome experimental limitations.

### Limitations and Outlook

There are several limitations in our methodological approach. First, we used independent component analysis to derive whole-brain functional networks at different scales. As with any other higher-order statistical method, independent component analysis is not free of underlying assumptions and specifically assumes maximal spatial independence of the networks (Jutten and Herault, 1991). Future studies could consider additional analyses using other metrics such as network measures. However, as Arslan et al. (2018) and Hilger et al. (2020) demonstrated in their studies (Arslan et al., 2018; Hilger et al., 2020), many network measures are largely altered by the spatial scale and appropriate correction techniques should be used for such analyses across scales.

Second, our analysis was focused on spatial scales of the brain dynamics at the macroscale. For investigations of microscale brain dynamics, other spatial and temporal scales might be relevant. Therefore, future studies could consider exploring brain dynamics of cellular-level networks using microscale imaging tools such as optical imaging. Ideally analyses of brain dynamics should bridge macro- and microscales; with corresponding methods currently under investigation (Weiskopf et al., 2015; Larivière et al., 2019; Gao et al., 2020).

Third, both the estimation of whole-brain functional networks as well as the calculation of the entropy of the network transition activity was done on a group level and during rest. Future studies could compare the entropy of network transitions on an individual level and under consideration of behavior and cognition, relating individual cognition to dynamical behavior of brain networks. For such approaches, vector-based instead of a region-based parcellations might account for better individual variations (Liu et al., 2021).

Overall, our results suggest that whole-brain functional brain networks operate at an optimum of about 300 regions and a timescale of about 150 ms. We contribute to the understanding of the dynamical behavior of whole-brain networks, which could inspire future human neuroimaging studies to harmonize spatiotemporal scales and neuroimaging modalities and use dynamical models to create connections between micro- and macroscopic scales.

## Data Availability Statement

Publicly available datasets were analyzed in this study. This data can be found here: db.humanconnectome.org.

## Ethics Statement

The studies involving human participants were reviewed and approved by Ethical Committee of the Human Connectome Project. The patients/participants provided their written informed consent to participate in this study.

## Author Contributions

XK, MK, and GD contributed to the conception, design, analysis, and data interpretation and drafted and critically revised the manuscript. AL-G contributed to the analysis, manuscript drafting, and revision of the manuscript. All authors contributed to the article and approved the submitted version.

## Conflict of Interest

The authors declare that the research was conducted in the absence of any commercial or financial relationships that could be construed as a potential conflict of interest.

## Publisher’s Note

All claims expressed in this article are solely those of the authors and do not necessarily represent those of their affiliated organizations, or those of the publisher, the editors and the reviewers. Any product that may be evaluated in this article, or claim that may be made by its manufacturer, is not guaranteed or endorsed by the publisher.

## Footnotes

1
http://dsi-studio.labsolver.org

## References

[B1] Achard S. Salvador R. Whitcher B. Suckling J. Bullmore E. (2006). A resilient, low-frequency, small-world human brain functional network with highly connected association cortical hubs. *J. Neurosci.* 26 63–72. 10.1523/jneurosci.3874-05.2006 16399673 PMC6674299

[B2] Alexandrov Y. I. (1999). “Physiological regularities of the dynamics of individual experience and the “stream of consciousness,” in *Neural Bases and Psychological Aspects of Consciousness*, eds Teddei-Ferretti C. Musio C. (Singapore: World Scientific), 201. 10.1142/9789814313254_0017

[B3] Amico E. Goñi J. (2018). The quest for identifiability in human functional connectomes. *Sci. Rep.* 8:8254. 29844466 10.1038/s41598-018-25089-1 PMC5973945

[B4] Arslan S. Ktena S. I. Makropoulos A. Robinson E. C. Rueckert D. Parisot S. (2018). Human brain mapping: a systematic comparison of parcellation methods for the human cerebral cortex. *Neuroimage* 170 5–30. 10.1016/j.neuroimage.2017.04.014 28412442

[B5] Ashburner J. (2007). A fast diffeomorphic image registration algorithm. *Neuroimage* 38 95–113. 10.1016/j.neuroimage.2007.07.007 17761438

[B6] Ashourvan A. Gu S. Mattar M. G. Vettel J. M. Bassett D. S. (2017). The energy landscape underpinning module dynamics in the human brain connectome. *Neuroimage* 157 364–380. 10.1016/j.neuroimage.2017.05.067 28602945 PMC5600845

[B7] Baker A. P. Brookes M. J. Rezek I. A. Smith S. M. Behrens T. Probert Smith P. J. (2014). Fast transient networks in spontaneous human brain activity. *Elife* 3:e01867. 24668169 10.7554/eLife.01867 PMC3965210

[B8] Bassett D. S. Meyer-Lindenberg A. Achard S. Duke T. Bullmore E. (2006). Adaptive reconfiguration of fractal small-world human brain functional networks. *Proc. Natl. Acad. Sci. U.S.A.* 103 19518–19523. 10.1073/pnas.0606005103 17159150 PMC1838565

[B9] Bassett D. S. Wymbs N. F. Porter M. A. Mucha P. J. Carlson J. M. Grafton S. T. (2011). Dynamic reconfiguration of human brain networks during learning. *Proc. Natl. Acad. Sci.U.S.A.* 108 7641–7646. 10.1073/pnas.1018985108 21502525 PMC3088578

[B10] Britz J. Van De Ville D. Michel C. M. (2010). BOLD correlates of EEG topography reveal rapid resting-state network dynamics. *Neuroimage* 52 1162–1170. 10.1016/j.neuroimage.2010.02.052 20188188

[B11] Brookes M. J. Tewarie P. K. Hunt B. A. E. Robson S. E. Gascoyne L. E. Liddle E. B. (2016). A multi-layer network approach to MEG connectivity analysis. *Neuroimage* 132 425–438. 10.1016/j.neuroimage.2016.02.045 26908313 PMC4862958

[B12] Cornblath E. J. Ashourvan A. Kim J. Z. Betzel R. F. Ciric R. Adebimpe A. (2020). Temporal sequences of brain activity at rest are constrained by white matter structure and modulated by cognitive demands. *Commun. Biol.* 3:261. 32444827 10.1038/s42003-020-0961-x PMC7244753

[B13] Craddock R. C. James G. A. Holtzheimer P. E. Hu X. P. Mayberg H. S. (2011). A whole brain fMRI atlas generated via spatially constrained spectral clustering. *Hum. Brain Mapp.* 33 1914–1928. 10.1002/hbm.21333 21769991 PMC3838923

[B14] Dadi K. Rahim M. Abraham A. Chyzhyk D. Milham M. Thirion B. (2019). Benchmarking functional connectome-based predictive models for resting-state fMRI. *Neuroimage* 192 115–134. 10.1016/j.neuroimage.2019.02.062 30836146

[B15] Deco G. Cabral J. Woolrich M. W. Stevner A. B. A. van Hartevelt T. J. Kringelbach M. L. (2017a). Single or multiple frequency generators in on-going brain activity: a mechanistic whole-brain model of empirical MEG data. *Neuroimage* 152 538–550. 10.1016/j.neuroimage.2017.03.023 28315461 PMC5440176

[B16] Deco G. Kringelbach M. L. Jirsa V. K. Ritter P. (2017b). The dynamics of resting fluctuations in the brain: metastability and its dynamical cortical core. *Sci. Rep.* 7:3095. 28596608 10.1038/s41598-017-03073-5 PMC5465179

[B17] Deco G. Cruzat J. Kringelbach M. L. (2019). Brain songs framework used for discovering the relevant timescale of the human brain. *Nat. Commun.* 10:583. 30718478 10.1038/s41467-018-08186-7 PMC6361902

[B18] Deco G. Kringelbach M. L. (2017). Hierarchy of information processing in the brain: a novel ‘intrinsic ignition’ framework. *Neuron* 94 961–968. 10.1016/j.neuron.2017.03.028 28595052

[B19] Deco G. Ponce-Alvarez A. Hagmann P. Romani G. L. Mantini D. Corbetta M. (2014). How local excitation–inhibition ratio impacts the whole brain dynamics. *J. Neurosci.* 34 7886–7898. 10.1523/jneurosci.5068-13.2014 24899711 PMC4044249

[B20] Deco G. Ponce-Alvarez A. Mantini D. Romani G. L. Hagmann P. Corbetta M. (2013). Resting-state functional connectivity emerges from structurally and dynamically shaped slow linear fluctuations. *J. Neurosci.* 33 11239–11252. 10.1523/jneurosci.1091-13.2013 23825427 PMC3718368

[B21] Engel A. K. Fries P. Singer W. (2001). Dynamic predictions: oscillations and synchrony in top-down processing. *Nat. Rev. Neurosci.* 2 704–716. 10.1038/35094565 11584308

[B22] Fan L. Zhong Q. Qin J. Li N. Su J. Zeng L. (2021). Brain parcellation driven by dynamic functional connectivity better capture intrinsic network dynamics. *Hum. Brain Mapp.* 42 1416–1433. 10.1002/hbm.25303 33283954 PMC7927310

[B23] Fornito A. Zalesky A. Bullmore E. T. (2010). Network scaling effects in graph analytic studies of human resting-state fMRI data. *Front. Syst. Neurosci.* 4:22. 10.3389/fnsys.2010.00022 20592949 PMC2893703

[B24] Gao R. van den Brink R. L. Pfeffer T. Voytek B. (2020). Neuronal timescales are functionally dynamic and shaped by cortical microarchitecture. *Elife* 9:e61277. 33226336 10.7554/eLife.61277 PMC7755395

[B25] Gerstner W. Sprekeler H. Deco G. (2012). Theory and simulation in neuroscience. *Science* 338 60–65. 10.1126/science.1227356 23042882

[B26] Glasser M. F. Sotiropoulos S. N. Wilson J. A. Coalson T. S. Fischl B. Andersson J. L. (2013). The minimal preprocessing pipelines for the human connectome project. *Neuroimage* 80 105–124. 10.1016/j.neuroimage.2013.04.127 23668970 PMC3720813

[B27] Glerean E. Salmi J. Lahnakoski J. M. Jääskeläinen I. P. Sams M. (2012). Functional magnetic resonance imaging phase synchronization as a measure of dynamic functional connectivity. *Brain Connect.* 2 91–101. 10.1089/brain.2011.0068 22559794 PMC3624768

[B28] Hilger K. Fukushima M. Sporns O. Fiebach C. J. (2020). Temporal stability of functional brain modules associated with human intelligence. *Hum. Brain Mapp.* 41 362–372. 10.1002/hbm.24807 31587450 PMC7267930

[B29] Honey C. J. Kötter R. Breakspear M. Sporns O. (2007). Network structure of cerebral cortex shapes functional connectivity on multiple time scales. *Proc. Natl. Acad. Sci.U.S.A.* 104 10240–10245. 10.1073/pnas.0701519104 17548818 PMC1891224

[B30] Horn A. Blankenburg F. (2016). Toward a standardized structural–functional group connectome in MNI space. *Neuroimage* 124 310–322. 10.1016/j.neuroimage.2015.08.048 26327244

[B31] Horn A. Li N. Dembek T. A. Kappel A. Boulay C. Ewert S. (2018). Lead-DBS v2: towards a comprehensive pipeline for deep brain stimulation imaging. *Neuroimage* 184 293–316. 10.1016/j.neuroimage.2018.08.068 30179717 PMC6286150

[B32] Horn A. Reich M. Vorwerk J. Li N. Wenzel G. Fang Q. (2017). Connectivity predicts deep brain stimulation outcome in parkinson disease. *Ann. Neurol.* 82 67–78. 10.1002/ana.24974 28586141 PMC5880678

[B33] Jutten C. Herault J. (1991). Blind separation of sources, part I: an adaptive algorithm based on neuromimetic architecture. *Signal Process.* 24 1–10. 10.1016/0165-1684(91)90079-x

[B34] Koenig T. Prichep L. Lehmann D. Sosa P. V. Braeker E. Kleinlogel H. (2002). Millisecond by millisecond, year by year: normative EEG microstates and developmental stages. *Neuroimage* 16 41–48. 10.1006/nimg.2002.1070 11969316

[B35] Kreher B. W. Mader I. Kiselev V. G. (2008). Gibbs tracking: a novel approach for the reconstruction of neuronal pathways. *Magn. Reson. Med.* 60 953–963. 10.1002/mrm.21749 18816816

[B36] Kumar S. Yoo K. Rosenberg M. D. Scheinost D. Constable R. T. Zhang S. (2019). An information network flow approach for measuring functional connectivity and predicting behavior. *Brain Behav.* 9:e01346. 31286688 10.1002/brb3.1346 PMC6710195

[B37] Larivière S. Vos de Wael R. Paquola C. Hong S.-J. Mišić B. Bernasconi N. (2019). Microstructure-informed connectomics: enriching large-scale descriptions of healthy and diseased brains. *Brain Connect.* 9 113–127. 10.1089/brain.2018.0587 30079754 PMC6444904

[B38] Liégeois R. Li J. Kong R. Orban C. Van De Ville D. Ge T. (2019). Resting brain dynamics at different timescales capture distinct aspects of human behavior. *Nat. Commun.* 10:2317. 31127095 10.1038/s41467-019-10317-7 PMC6534566

[B39] Liu M. Liu X. Hildebrandt A. Zhou C. (2020). Individual cortical entropy profile: test–retest reliability, predictive power for cognitive ability, and neuroanatomical foundation. *Cereb. Cortex Commun.* 1:tgaa015. 34296093 10.1093/texcom/tgaa015 PMC8153045

[B40] Liu R. Li M. Dunson D. B. (2021). PPA: principal parcellation analysis for brain connectomes and multiple traits. *ArXiv* [Preprint] 10.1101/256933 ArXiv: 210303478, 37286151

[B41] Lopes-dos-Santos V. Ribeiro S. Tort A. B. L. (2013). Detecting cell assemblies in large neuronal populations. *J. Neurosci. Methods* 220 149–166. 10.1016/j.jneumeth.2013.04.010 23639919

[B42] Lungarella M. Sporns O. (2006). Mapping information flow in sensorimotor networks. *PLoS Comput. Biol.* 2:e144. 10.1371/journal.pcbi.0020144 17069456 PMC1626158

[B43] Mai A.-T. Grootswagers T. Carlson T. A. (2019). In search of consciousness: examining the temporal dynamics of conscious visual perception using MEG time-series data. *Neuropsychologia* 129 310–317. 10.1016/j.neuropsychologia.2019.04.015 31028755

[B44] Marèenko V. A. Pastur L. (1967). Distribution of eigenvalues for some sets of random matrices. *Math. USSR Sb.* 1 457–483. 10.1070/sm1967v001n04abeh001994

[B45] Mashour G. A. Hudetz A. G. (2018). Neural correlates of unconsciousness in large-scale brain networks. *Trends Neurosci.* 41 150–160. 10.1016/j.tins.2018.01.003 29409683 PMC5835202

[B46] Messé A. (2019). Parcellation influence on the connectivity-based structure–function relationship in the human brain. *Hum. Brain Mapp.* 41 1167–1180. 10.1002/hbm.24866 31746083 PMC7267927

[B47] Michel C. M. Koenig T. (2018). EEG microstates as a tool for studying the temporal dynamics of whole-brain neuronal networks: a review. *Neuroimage* 180 577–593. 10.1016/j.neuroimage.2017.11.062 29196270

[B48] Musso F. Brinkmeyer J. Mobascher A. Warbrick T. Winterer G. (2010). Spontaneous brain activity and EEG microstates. A novel EEG/fMRI analysis approach to explore resting-state networks. *Neuroimage* 52 1149–1161. 10.1016/j.neuroimage.2010.01.093 20139014

[B49] Niu Y. Wang B. Zhou M. Xue J. Shapour H. Cao R. (2018). Dynamic complexity of spontaneous BOLD activity in Alzheimer’s disease and mild cognitive impairment using multiscale entropy analysis. *Front. Neurosci.* 12:677. 10.3389/fnins.2018.00677 30327587 PMC6174248

[B50] Peyrache A. Benchenane K. Khamassi M. Wiener S. I. Battaglia F. P. (2010). Principal component analysis of ensemble recordings reveals cell assemblies at high temporal resolution. *J. Comput. Neurosci.* 29 309–325. 10.1007/s10827-009-0154-6 19529888 PMC2940043

[B51] Proix T. Spiegler A. Schirner M. Rothmeier S. Ritter P. Jirsa V. K. (2016). How do parcellation size and short-range connectivity affect dynamics in large-scale brain network models? *Neuroimage* 142 135–149. 10.1016/j.neuroimage.2016.06.016 27480624

[B52] Rämö P. Kauffman S. Kesseli J. Yli-Harja O. (2007). Measures for information propagation in Boolean networks. *Phys. Nonlinear Phenom.* 227 100–104. 10.1016/j.physd.2006.12.005

[B53] Saenger V. M. Kahan J. Foltynie T. Friston K. Aziz T. Z. Green A. L. (2017). Uncovering the underlying mechanisms and whole-brain dynamics of deep brain stimulation for Parkinson’s disease. *Sci. Rep.* 7:9882. 28851996 10.1038/s41598-017-10003-y PMC5574998

[B54] Salti M. Monto S. Charles L. King J.-R. Parkkonen L. Dehaene S. (2015). Distinct cortical codes and temporal dynamics for conscious and unconscious percepts. *Elife* 4:e05652. 10.7554/eLife.05652 PMC4467230 25997100

[B55] Schaefer A. Kong R. Gordon E. M. Laumann T. O. Zuo X.-N. Holmes A. J. (2018). Local-global parcellation of the human cerebral cortex from intrinsic functional connectivity MRI. *Cereb. Cortex* 28 3095–3114. 10.1093/cercor/bhx179 28981612 PMC6095216

[B56] Setsompop K. Kimmlingen R. Eberlein E. Witzel T. Cohen-Adad J. McNab J. A. (2013). Pushing the limits of in vivo diffusion MRI for the human connectome project. *Neuroimage* 80 220–233. 10.1016/j.neuroimage.2013.05.078 23707579 PMC3725309

[B57] Shannon C. E. (1948). A mathematical theory of communication. *Bell Syst. Tech. J.* 27 379–423.

[B58] Shew W. L. Yang H. Yu S. Roy R. Plenz D. (2011). Information capacity and transmission are maximized in balanced cortical networks with neuronal avalanches. *J. Neurosci.* 31 55–63. 10.1523/jneurosci.4637-10.2011 21209189 PMC3082868

[B59] Stephan K. E. Weiskopf N. Drysdale P. M. Robinson P. A. Friston K. J. (2007). Comparing hemodynamic models with DCM. *Neuroimage* 38 387–401. 10.1016/j.neuroimage.2007.07.040 17884583 PMC2636182

[B60] Stitt I. Hollensteiner K. J. Galindo-Leon E. Pieper F. Fiedler E. Stieglitz T. (2017). Dynamic reconfiguration of cortical functional connectivity across brain states. *Sci. Rep.* 7:8797. 28821753 10.1038/s41598-017-08050-6 PMC5562766

[B61] Tagliazucchi E. Balenzuela P. Fraiman D. Chialvo D. R. (2012). Criticality in large-scale brain FMRI dynamics unveiled by a novel point process analysis. *Front. Physiol.* 3:15. 10.3389/fphys.2012.00015 22347863 PMC3274757

[B62] Tang Y.-Y. Rothbart M. K. Posner M. I. (2012). Neural correlates of establishing, maintaining, and switching brain states. *Trends Cogn. Sci.* 16 330–337. 10.1016/j.tics.2012.05.001 22613871 PMC3419378

[B63] Thompson G. J. Magnuson M. E. Merritt M. D. Schwarb H. Pan W.-J. McKinley A. (2013). Short-time windows of correlation between large-scale functional brain networks predict vigilance intraindividually and interindividually. *Hum. Brain Mapp.* 34 3280–3298. 10.1002/hbm.22140 22736565 PMC6870033

[B64] Van De Ville D. Britz J. Michel C. M. (2010). EEG microstate sequences in healthy humans at rest reveal scale-free dynamics. *Proc. Natl. Acad. Sci. U.S.A.* 107 18179–18184. 10.1073/pnas.1007841107 20921381 PMC2964192

[B65] van der Meer J. N. Breakspear M. Chang L. J. Sonkusare S. Cocchi L. (2020). Movie viewing elicits rich and reliable brain state dynamics. *Nat. Commun.* 11:5004. 33020473 10.1038/s41467-020-18717-w PMC7536385

[B66] Van Essen D. C. Smith S. M. Barch D. M. Behrens T. E. J. Yacoub E. Ugurbil K. (2013). The WU-minn human connectome project: an overview. *Neuroimage* 80 62–79. 10.1016/j.neuroimage.2013.05.041 23684880 PMC3724347

[B67] Vidaurre D. Smith S. M. Woolrich M. W. (2017). Brain network dynamics are hierarchically organized in time. *Proc. Natl. Acad. Sci. U.S.A.* 114 12827–12832. 29087305 10.1073/pnas.1705120114 PMC5715736

[B68] Wang D. J. J. Jann K. Fan C. Qiao Y. Zang Y.-F. Lu H. (2018). Neurophysiological basis of multi-scale entropy of brain complexity and its relationship with functional connectivity. *Front. Neurosci.* 12:352. 10.3389/fnins.2018.00352 29896081 PMC5986880

[B69] Weiskopf N. Mohammadi S. Lutti A. Callaghan M. F. (2015). Advances in MRI-based computational neuroanatomy: from morphometry to in-vivo histology. *Curr. Opin. Neurol.* 28 313–322. 10.1097/wco.0000000000000222 26132532

[B70] Wildie M. Shanahan M. (2012). Metastability and chimera states in modular delay and pulse-coupled oscillator networks. *Chaos Interdiscip. J. Nonlinear Sci.* 22:043131. 10.1063/1.4766592 23278066

[B71] Wilson H. R. Cowan J. D. (1972). Excitatory and inhibitory interactions in localized populations of model neurons. *Biophys. J.* 12 1–24. 10.1016/s0006-3495(72)86068-5 4332108 PMC1484078

[B72] Wong K.-F. Wang X.-J. (2006). A recurrent network mechanism of time integration in perceptual decisions. *J. Neurosci.* 26 1314–1328. 10.1523/jneurosci.3733-05.2006 16436619 PMC6674568

[B73] Wutz A. Weisz N. Braun C. Melcher D. (2014). Temporal windows in visual processing: “prestimulus brain state” and “poststimulus phase reset” segregate visual transients on different temporal scales. *J. Neurosci.* 34 1554–1565. 10.1523/jneurosci.3187-13.2014 24453342 PMC3898303

[B74] Yeo B. T. Krienen F. M. Sepulcre J. Sabuncu M. R. Lashkari D. Hollinshead M. (2011). The organization of the human cerebral cortex estimated by intrinsic functional connectivity. *J. Neurophysiol.* 106 1125–1165. 10.1152/jn.00338.2011 21653723 PMC3174820

[B75] Yoo H. B. Moya B. E. Filbey F. M. (2020). Dynamic functional connectivity between nucleus accumbens and the central executive network relates to chronic cannabis use. *Hum. Brain Mapp.* 41 3637–3654. 10.1002/hbm.25036 32432821 PMC7416060

[B76] Yourganov G. Chen X. Lukic A. S. Grady C. L. Small S. L. Wernick M. N. (2011). Dimensionality estimation for optimal detection of functional networks in BOLD fMRI data. *Neuroimage* 56 531–543. 10.1016/j.neuroimage.2010.09.034 20858546 PMC3052418

[B77] Yuan H. Zotev V. Phillips R. Drevets W. C. Bodurka J. (2012). Spatiotemporal dynamics of the brain at rest–exploring EEG microstates as electrophysiological signatures of BOLD resting state networks. *Neuroimage* 60 2062–2072. 10.1016/j.neuroimage.2012.02.031 22381593

[B78] Yuan J. Li X. Zhang J. Luo L. Dong Q. Lv J. (2018). Spatio-temporal modeling of connectome-scale brain network interactions via time-evolving graphs. *Neuroimage* 180 350–369. 10.1016/j.neuroimage.2017.10.067 29102809 PMC5943195

