# Supplementary material for: Revealing the Relevant Spatiotemporal Scale Underlying Whole-Brain Dynamics
Source: Front Neurosci. 2021 Oct 22;15:715861. doi: 10.3389/fnins.2021.715861 (PMC8569182; doi:10.3389/fnins.2021.715861)
Supplement: Supplementary file 1 [file Data_Sheet_1.docx]

**Supplementary Files**

**
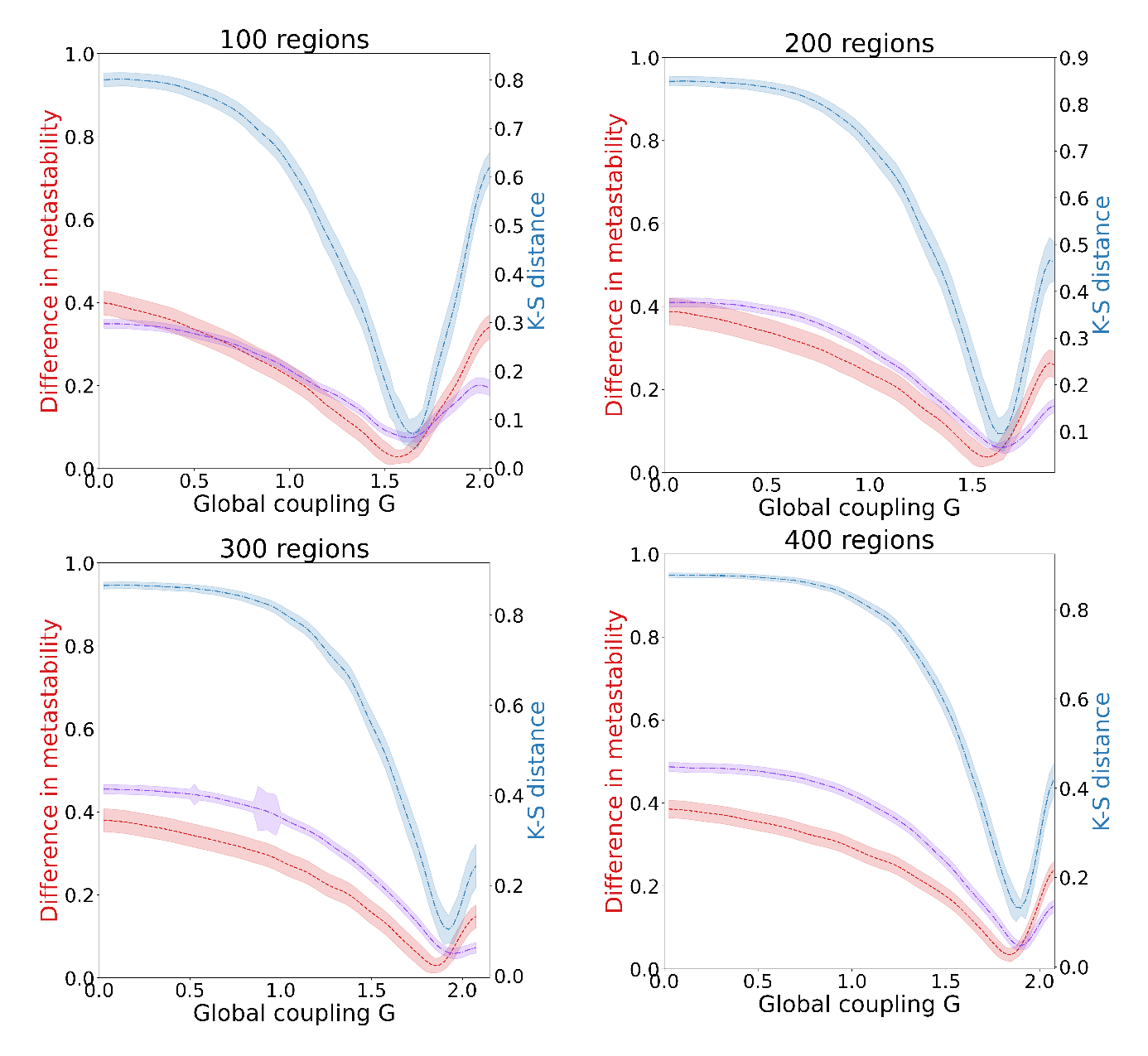
**

**Figure S1. Fitting of the optimal working point of the model to the dynamical characteristics of the empirical data.** For the estimation of the global coupling value G, we used two different metrics for model fitting representing different aspects of brain dynamics. We compared the difference of metastability (mean and standard deviations in red), the Kolmogorov-Smirnov distance of phase similarity matrix distributions (mean and standard deviations in blue) and of functional connectivity dynamics (mean and standard deviations in blue in purple) across the four spatial scales (depicted in different colors in each panel) and chose G according to the minima of both metrics.


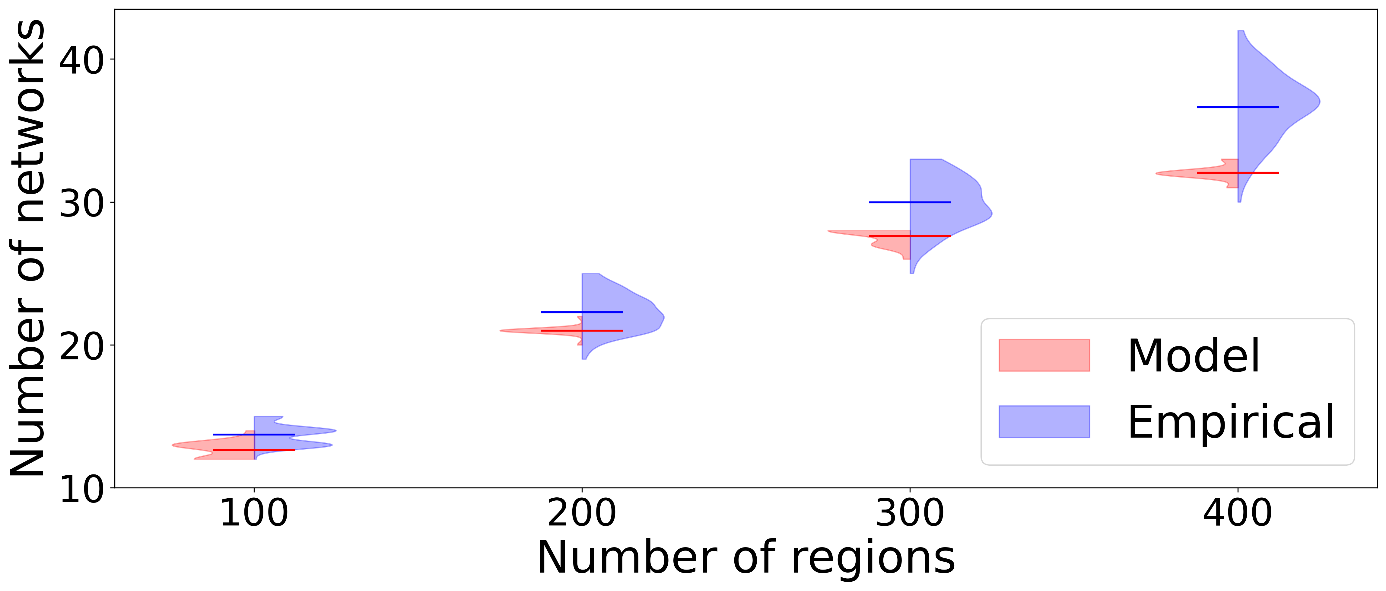


**Figure S2. Number of functional networks derived from simulated time series (in red) and empirical time series (in blue) at a TR = 720 ms.** As can be seen in the figure, we were able to retrieve comparable numbers of functional networks from the simulated time series and empirical time series and a similar increase in numbers was observed across spatial scales. Means are displayed by the horizontal lines in each violin plots. Each datapoint depicts a random group of 10 subjects in the empirical data or a simulation trial simulating a group of 10 subjects.

| **Name** | **Value** | **Unit** | **Description** |
| --- | --- | --- | --- |
| *G* | Determined by model fitting |  | Global coupling value |
| *C* | Determined by structural connectivity matrix |  | Connection weights |
| $I_{thr}^{\left( E \right)}$ | 0.403 | nA | Excitatory threshold current |
| $I_{thr}^{\left( I \right)}$ | 0.288 | nA | Inhibitory threshold current |
| $\tau_{NMDA}$ | 0.1 | s | decay time constant (NMDA) |
| $\tau_{GABA}$ | 0.01 | s | decay time constant (GABA) |
| $I_{0}$ | 0.382 | nA |  |
| $w_{E}$ | 1 | - | weight of the excitatory input |
| $w_{I}$ | 0.7 | - | weight of the inhibitory input |
| $w_{+}$ | 1.4 | - | weight of the recurrent excitation |
| $w_{NMDA}$ | 0.15 | - | Weight of excitatory synaptic coupling |
| $w_{n}$ | Determined by feedback inhibition control mechanism | - | Weight of feedback inhibition |

**Table S1: Parameters of the DMF model.** All parameters are taken from previous publications (Deco et al. 2013, 2014).
